# A mathematical model to predict the risk of hepatitis B infection through needle/syringe sharing in mass vaccination

**DOI:** 10.1186/2049-9957-2-28

**Published:** 2013-11-19

**Authors:** Etsuji Okamoto

**Affiliations:** 1 Department of Health and Welfare Service Research, National Institute of Public Health, Saitama 351-0197 2-3-6 Minami, Wako-shi, Japan

**Keywords:** Hepatitis, Mass tuberculin skin test, Needle sharing, Mathematical model, Iatrogenic infection

## Abstract

**Background:**

The Japanese Government settled a class litigation case with hepatitis B virus (HBV) carriers who claim to have been infected through needle/syringe sharing in childhood mass vaccination with a blanket compensation agreement. However, it is difficult to estimate how many of the present HBV carriers were infected horizontally from mass vaccination and how many were infected vertically from mothers.

**Methods:**

A mathematical model to predict the risk of infection through needle/syringe sharing in mass vaccination was proposed and a formula was developed. The formula was presented in a logarithmic graph enabling users to estimate how many people will be infected if a needle/syringe is shared by how many people for how many times under certain probability of infection. The formula was then applied to the historical data of mass tuberculin skin tests (TSTs) and BCG inoculation, from which a best estimate of how much needle/syringe sharing was practiced in different birth cohorts was determined.

**Results:**

For the oldest cohort born between 1951 and 1955, the prevalence of HBV carriers—0.65% at birth through vertical transmission—more than doubled in 1995 (1.46%) through horizontal transmission. If the probability of infection through needle/syringe sharing is assumed to be 10%, it is theoretically likely that an average of five or more people shared a needle/syringe four times to achieve the prevalence of HBV carriers in 1995. However, for the youngest cohort born between 1981 and 1985, the effects of needle/syringe sharing were negligible because the later prevalence of HBV carriers was lower than the prevalence at birth.

**Conclusions:**

More than half of the HBV carriers born in the early 1950s might have contracted the disease by mass vaccinations. Japan’s experience needs to be shared with other countries as a caution in conducting mass vaccination programs under scarce needle/syringe supply (291 words).

## Multilingual abstracts

Please see Additional file 1 for translations of the abstract into the six official working languages of the United Nations.

## Background

Historically, Japan, as well as most Asian countries, has a high prevalence of hepatitis B and C virus (HBV, HCV) carriers [1]. In 1989, five HBV carriers sued the Government claiming that they had been infected with the virus through needle/syringe sharing in childhood mass vaccination [2]. In 2002, another group of HCV carriers also filed a class litigation case against the Government and pharmaceutical manufacturers claiming that they had been infected iatrogenically through tainted blood products (fibrinogen) [3].

In 2008, the Government accepted an out-of-court settlement with the HCV plaintiffs setting the precedents of damages (e.g., 36 million yen [approximately $US 370,000] for deaths, liver cancer, and cirrhosis caused by HCV) [4]. The Government accepted another settlement with the HBV plaintiffs in June 2011 with a blanket compensation agreement: Awarding the same damages to all HBV carriers who had been infected through needle/syringe sharing in childhood mass vaccination [5].

The potential financial burden of the blanket compensation could be gigantic given the high prevalence of HBV among the Japanese population, and the unknown prospect of how many were infected from needle/syringe sharing in mass vaccination and how many were not. Since almost the entire Japanese population was vaccinated in childhood before 1988, when syringe sharing was officially banned (needle sharing was banned for tuberculin skin test (TST) in 1950 [6] and for mass vaccination in 1958 [7]), the total amount of damages could reach up to 3.2 trillion yen ($US 32 billion, estimates by Ministry of Health, Labour and Welfare) if all HBV carriers are entitled to compensation.

The settlement with the HBV plaintiffs included a mandate that the Government investigate the extent of needle/syringe sharing at that time, and the reason why such risky practice was left uncontrolled. A research group was organized to conduct questionnaire surveys with municipal governments and interviewed retired public health officers about how mass vaccinations were conducted at that time. The findings were compiled as a 400-page report [8]. Unfortunately, the results—not surprisingly—were disappointing. The research group sent questionnaire to 1,701 municipal governments and received 1,149 replies (67.5%), of which only 11 replied that they kept records of mass vaccinations from 1962 or before, and only four for 1954 or before (the rest replied that they had no records from those times). The research group also sent questionnaire to 61 retired directors of public health centers and only 37 responded (60.7%). Of the 33 who responded to the question on whether they instructed needle/syringe exchange, 17 did not reply or replied that they had no memory. Eleven replied that they had instructed that the needle/syringe must be exchanged individually, however five of them replied that they had not. One of the respondents apologized by writing “I am now 86 and suffering from a stroke. I cannot answer correctly about experiences that happened such a long time ago…”.

Hence, there is a need to develop a statistically sound model and a formula to predict how many of the current HBV carriers are considered to have been infected through needle/syringe sharing in mass vaccination. Since it is difficult to demonstrate with evidence how needle/syringe sharing was practiced such a long time ago, one can only rely on mathematical modeling using historical data.

## Methods

### Ethical consideration

Ethical approval was not sought because this study is a theoretical one using administrative data only.

### A mathematical model

The chance of having ***i*** infected persons in a group of ***n*** with a prevalence of ***p*** sharing a needle/syringe follows a binomial distribution: B(i;n,p). When there are ***i*** infected persons in a group of ***n***, the number of uninfected persons is ***n-i***.

The chances of an uninfected person receiving a shot BEFORE ***i*** infected persons (i.e., the chance of the uninfected person escaping the infection) is expressed as 1/(***i*** + 1) because there are a total of (***i*** + 1) persons including both the uninfected person and the ***i*** infected persons who have equal chances of receiving a shot ahead of others. Then, the chance of an uninfected person getting infected for receiving a shot AFTER any of infected persons is ***i***/(***i*** + 1).

**Figure 1:**
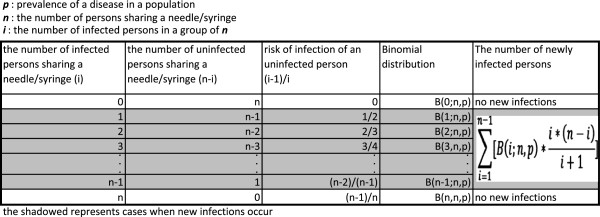
Illustration of a formula to predict the number of new infection.

New infections will not occur where none or all of the ***n*** persons sharing a needle/syringe are infected. New infections only occur when there is one or more (1 < =***i*** < =***n***-1) infected persons in a group of ***n*** sharing a needle/syringe. Hence, the expected number of people in a group of ***n*** being infected is the sum of B(i;n,p)*(n-i)*i/(i + 1) for 1 < =***i*** < =***n***-1. Note that the chance of getting infected depends on the number of infected persons, ***i***, in the group and is NOT related to the size of the group sharing a needle/syringe, ***n***. The logic is illustrated in Figure 1.

Let’s assume that an uninfected person gets infected from an infected person when a needle/syringe is shared by ***n*** people in a mass vaccination, and the uninfected person receives a shot AFTER the infected person with the probability ***r***. When such a mass vaccination is conducted in a population with a prevalence of the disease ***p***, the number of newly infected persons ***K*** is expressed as follows:

(1) $$K=r*\sum_{i=1}^{n-1}\left[B\left(i;n,p\right)*\frac{i*\left(n-i\right)}{i+1}\right]$$

Hence, the prevalence after a mass vaccination will be increased by ***K/n*** resulting in the new prevalence of ***p + K/n***.

### Graphical presentation

**Figure 2:**
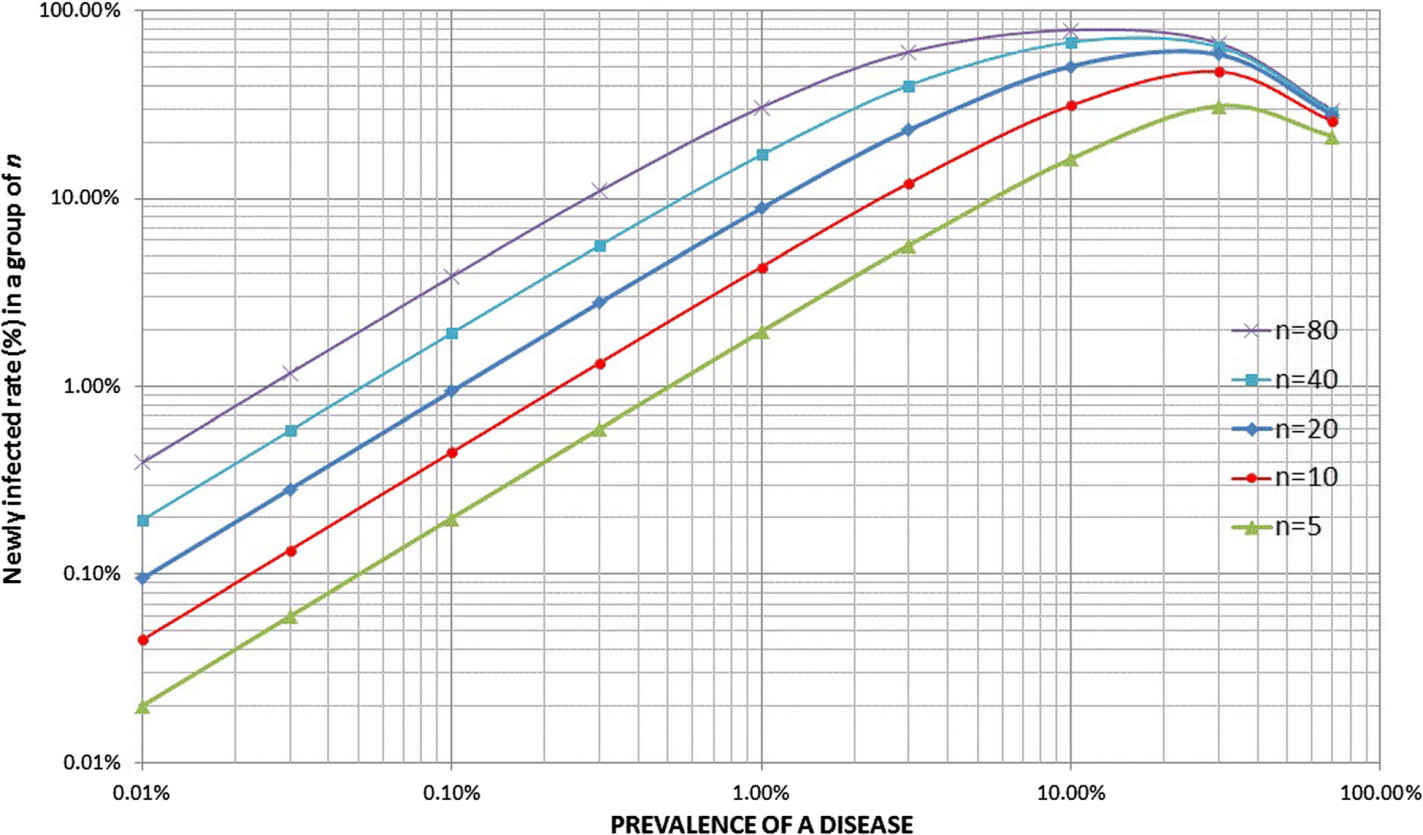
Graphical presentation of the formula to predict new infection rate through needle/syringe sharing.

The formula (1) is presented graphically with changing parameters: ***n*** and ***p*** where ***r*** is set at 1 (see Figure 2). The risk of a new infection increases as the prevalence and the number of people sharing a needle/syringe increases. In a population with prevalence of 1% of a disease, 2% of the population will be newly infected if a needle/syringe is shared by five people, resulting in 3% prevalence after a mass vaccination. If a needle/syringe is shared by 20 people, it will newly infect 9% of the population making the resultant prevalence of 10%. If the prevalence is 10% and a needle/syringe is shared by 80 people, it will infect 80% of the population, i.e., only 10% of the people will be left uninfected. However, once the prevalence of a disease exceeds a certain level, the number of new infections will be less because there will be fewer uninfected persons.

### Application of the mass TST and BCG inoculation program

The mass tuberculin skin test (TST) program was enforced pursuant to the Tuberculosis Control Act and almost every newborn baby received a TST. The formula (1) was applied to estimate the number of HBV carriers infected through needle/syringe sharing in mass TST with the following assumptions.

#### Mass TST and BCG inoculation program

**Figure 3:**
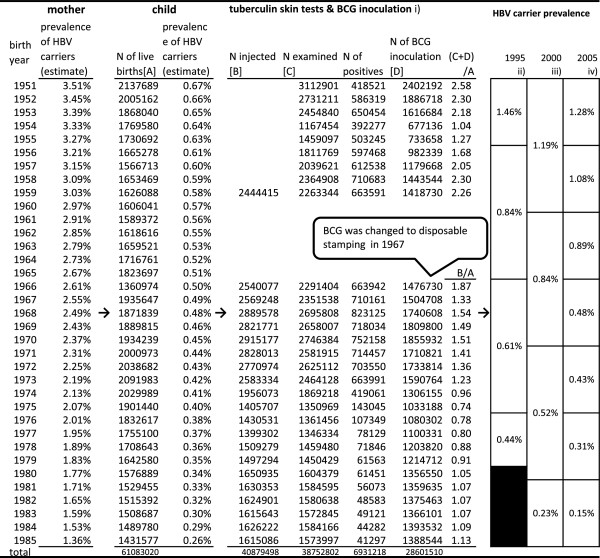
Prevalence of HBV carriers and mass tuberculin skin tests/BCG inoculation of children born 1951-85.

Control of tuberculosis (TB) was one of the most important public health issues in Japan, and mass TST and BCG inoculations on those with negative results have been conducted pursuant to the Tuberculosis Control Act. The data on age-specific (0–5 year olds) number of TST and BCG recipients were taken from the annual reports of public health centers from 1951 onwards (see Figure 3). The administrative reports summarized public health center activities, and data definition and collection procedures were consistent over the years. However, age groups were not so consistent. The data between 1960 and 1965 are aggregated in all ages, and children’s data were not available and hence excluded from analysis. Other vaccinations for small pox, diphtheria, pertussis, and typhoid fever were also responsible for HBV infection but they were not included in the analysis because age-specific data were not available over the years.

Since needle/syringe sharing was officially banned in January 1988 and the prevention program for HB mother-to-infant transmission was fully introduced in 1986, birth cohorts born between 1951 and 1985 were focused on.

Children with negative (plus quasi-negative) TST received BCG inoculations. On average, children born between 1951 and 1959 received two or more injections. The reports before 1959 do not provide the number of people injected. In 1967, BCG was changed to a disposable stamping method and the risk of iatrogenic infection through BCG was eliminated in children born in this year or later. Still, children born between 1966 and 1985 received, on average, one or more TST.

#### Prevalence of HBV carriers among mothers and pre-school children

Prevalence of HBV carriers (HBsAg(+)) among mothers and pre-school children are not known before a certain time and must be estimated. Since almost all HBV carriers are attributable to vertical (mother-to-infant) transmission and the risk is highest when mothers are HBeAg(+), in which case 85–90% of newborn babies will be HBV carriers [9], the prevalence of HBV carriers among newborn babies can be estimated when the prevalence of mothers can also be estimated.

Mori reported the prevalence of HBsAg(+) among pregnant mothers examined at Yokohama city public health centers (N = 18,152 or approximately 11% of pregnant mothers in the city) between 1976 and 1980 as 2.0% (358/18152) [10].

As well as that, the prevention program to eliminate mother-to-infant transmission of hepatitis B, introduced in 1986, provides a national estimate of the prevalence of HBV carriers among pregnant mothers and newborn babies [11]. Shiraki estimated the prevalence of HBV carriers among newborn babies born in 1985 as 0.26% by multiplying the prevalence of HBV carriers among pregnant mothers (1.36%) with the rate of HBeAg(+) (22.5%) and the probability of a newborn baby to become a carrier (85%) [12]. Theoretically, the prevalence of HBV carriers infected vertically through mother-to-infant transmission will be reduced to 20% (0.225*0.85 = 0.2) in every generation without any preventive measures. If the prevalence in later generations is higher than expected, it suggests augmentation through horizontal transmission such as mass vaccinations or other routes of infection.

**Figure 4:**
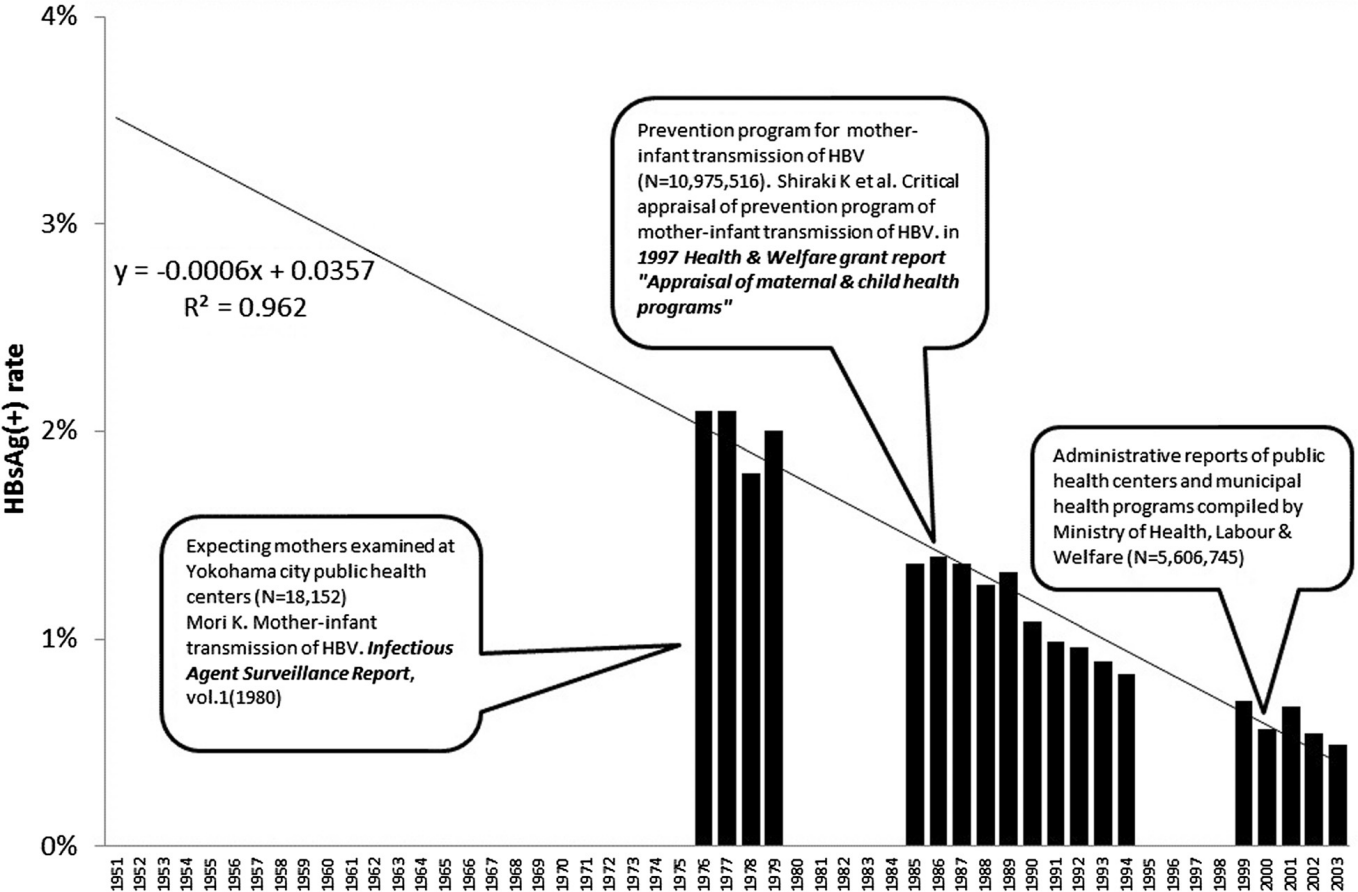
Prevalence of HBsAg (+) among pregnant mothers.

The author applied a trend line to all available data and obtained the following regression formula with high R^2^ (0.962) (see Figure 4). The prevalence of HBV carriers among pregnant mothers, ***W***, in the ***V***th year between 1951 and 1985 can be estimated by:

W=-0.0006*V+0.0357

This means that the prevalence of HBV carriers in 1951 was estimated to be 3.51% and decreases by 0.06% every year. The estimated prevalence among pregnant mothers in each year was converted into the prevalence among newborn babies by applying Shiraki’s formula. Based on these assumptions, the children born in 1951 were estimated to have the prevalence of HBV of 0.67%, or roughly one out of 150 was an HBV carrier.

#### Current cohort-specific prevalence of HBV carriers

Children who became HBV carriers may infect other children through mass TST and BCG inoculations, or other mass vaccinations such as smallpox, diphtheria, typhoid, paratyphoid, and pertussis, as well as by other means. Children infected in their pre-school age (0–5 years old) carry a high risk of becoming HBV carriers (the genotype of HBV prevalence in Japan has been predominantly type B or C, which do not cause asymptomatic carriers unless children under the age of six are infected. On the other hand, genotype A causes asymptomatic carriers even in adulthood. HBV carriers with genotype A(Ae) do not qualify for compensation because HBV of genotype A was not detected before 1996, and hence are not considered to have been infected through mass vaccination before 1988 [13]). Then, the prevalence of HBV carriers in later age should be higher than their prevalence after birth.

The author compiled three different sources estimating age-specific prevalence of HBV carriers in 1995, 2000, and 2005 [14-16]. The first two sources provide data of ten-year age groups and the last provides five-year age groups. These data are presented in Figure 3 in the order of birth cohorts.

The prevalence in 1995 was higher than the prevalence after birth in all cohorts. The discrepancy is wider in the older cohorts. For example, the cohort born between 1951 and 1955 was estimated to have the prevalence of 0.65% at birth. However, their prevalence in 1995 was 1.46% and then declined to 1.28% in 2005. The more than two-fold increase between birth and middle age is attributable to horizontal transmission either through mass vaccination or other routes. The subsequent decline of prevalence of HBV carriers in some cohorts between 1995 and 2005 could be explained by not only hepatitis-related deaths, but also successful viral eradication thanks to improved treatment such as interferon.

#### Application of the mathematical model

To apply the mathematical model expressed in the formula (1), one must postulate the probability of infection of HBV, ***r***. For this, the evidence from occupational exposure provides reference.

In case of accidental needle-stick injuries, the risk of developing serologic evidence of HBV infection was 37–62% if the needle is contaminated with blood that is both HBsAg and HBeAg positive (the risk is lower if the HBeAg is negative: 23–37%). On the other hand, the average incidence of anti-HCV seroconversion after accidental percutaneous exposure from an HCV-positive source is 1.8% (range:0–7%) and the average risk of HIV transmission after a percutaneous exposure to HIV-infected blood is 0.3% (95% C.I.: 0.2–0.5%) [17]. Obviously, the risk of HBV is far greater than HCV and HIV.

Accidental needle-stick injury includes hollow needle injury and injury during surgery involving massive blood exposure posing a high risk of transmission. On the other hand, BCG is injected subcutaneously or intracutaneously, and the amount of blood exposure by needle/syringe sharing is considered to be smaller than those of accidental needle-stick injuries. In view of this evidence, it would be safe to assume that the risk of needle/syringe sharing poses a greater risk than HCV and HIV, but lower than accidental needle-stick injuries. So, the author assumed the probability to be ***10%***, somewhere between the lower margin of accidental needle-stick injuries (23%), and the average risk of HCV and HIV (1.8% and 0.3%).

## Results

Results are summarized in Figure 3.

**Figure 5:**
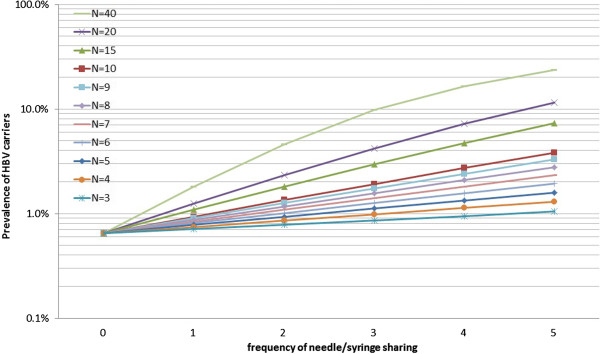
Prevalence of HBV carriers after repeated needle/syringe sharing.

In case of the oldest cohort (born between 1951 and 1955), with a median prevalence of 0.65% at birth, the prevalence of HBV carriers after repeated sharing of needle/syringe with the probability of infection **0.1** is illustrated in Figure 5. The average prevalence of this cohort in 1995 was 1.46%, and one can estimate how much needle/syringe sharing prevalence this cohort had “achieved” by locating a crossing of a horizontal graduation (somewhere between 1% and 2%), and curves of different average number of persons sharing a needle/syringe. For this particular cohort to achieve 1.46% prevalence, they are estimated to have either 1) shared a needle/syringe with more than 20 children one time, 2) more than 10 children two times, 3) more than seven children three times, or 4) more than five children four times.

Children of this cohort received TST and BCG inoculation two times on average ((C + D)/A in Figure 3). Considering other vaccinations, children in those days were likely to receive four vaccinations, and then the above estimation would tell that sharing a needle/syringe with as few as five persons would have resulted in the increased prevalence when they became middle aged.

In case of the youngest cohort (born between 1981 and 1985), with a median prevalence of 0.3% at birth, sharing the needle/syringe must have been negligible because their later prevalence declined: 0.23% in 2000 and 0.15% in 2005 (their 1995 data are not available because they were too young to donate blood at that time). This finding suggests that although the official ban of syringe sharing was 1988, sharing of needle/syringe was really abandoned at the beginning of the 1980s.

## Discussion

Large-scale settlements on HBV infection through mass vaccination give a false impression that all HBV infections were caused by needle/syringe sharing in mass vaccination. In fact, the mass-vaccination program simply augmented the prevalence of HBV carriers among children. Unless an accurate estimate of how many of HBV carriers were infected by mass vaccination is determined, it may lead to a denial of the mass-vaccination program and, more importantly, the contribution of public health activities.

Based on the agreements of the blanket compensation, all mass vaccinations before 27^th^ January 1988 are assumed to have been conducted with needle/syringe sharing. This was inevitable because it is difficult to demonstrate which mass vaccinations were conducted with needle/syringe sharing, and which were not.

However, this study suggests that needle/syringe sharing was negligible in young cohorts born after 1980 because the prevalence of HBV carriers when they grew up was lower than their estimated prevalence at birth. Although the official ban on needle/syringe sharing was belated to 1988, disposable needle/syringe was already common use by the middle of the 1970s. This means that the assumption that there was needle/syringe sharing in the 1980s cannot be justified by the evidence.

For the cohorts born between 1951 and 1955, there was evidence that the prevalence of carrying HBV in adulthood more than doubled than the prevalence at birth. It is possible that more than half of the HBV carriers of this cohort were infected through needle/syringe sharing. If the probability of infection of HBV through needle/syringe sharing is assumed to be 10%, it was demonstrated that sharing a needle/syringe by five or more children four times would have been enough to achieve their observed prevalence in 1995. This finding will also serve as evidence on how much needle/syringe sharing was practiced in mass vaccination. Three witnesses testified in the landmark lawsuit filed in June 1989 in Hokkaido [18]. A public health nurse who worked between 1951 and 1971 testified that one needle/syringe had been shared by eight people for TST and BCG inoculations (BCG was inoculated by way of intradermal injection before 1967), and by ten people for typhoid fever and paratyphoid vaccinations. Another public health nurse who worked between 1962 and 1995 testified that 14–15 people shared a needle/syringe for TST, 15–16 people for influenza vaccination, and 7–8 people for diphtheria and pertussis vaccinations. She also testified that disposable needle/syringes were in common use by 1981. A medical doctor who worked for a Sapporo city public health center testified that he had injected 5–6 people without changing the needle/syringe for TST, and added that all needle/syringes had been replaced with disposable ones by 1975 in all public health centers in Sapporo. The court determined that sharing of a needle/syringe was common until 1969 or 1970, and upheld the plaintiff’s claims.

Some municipalities departed from mass vaccination to individual vaccination. The city of Kobe introduced individual vaccinations as early as June 1969 by contracting out the practice to the local medical association [19]. This evidence coincides with the finding of this study that the gap between the prevalence of HBV carriers at birth and prevalence later in life was narrowed after the cohorts born in the mid-1970s.

### Practical implications

**Figure 6:**
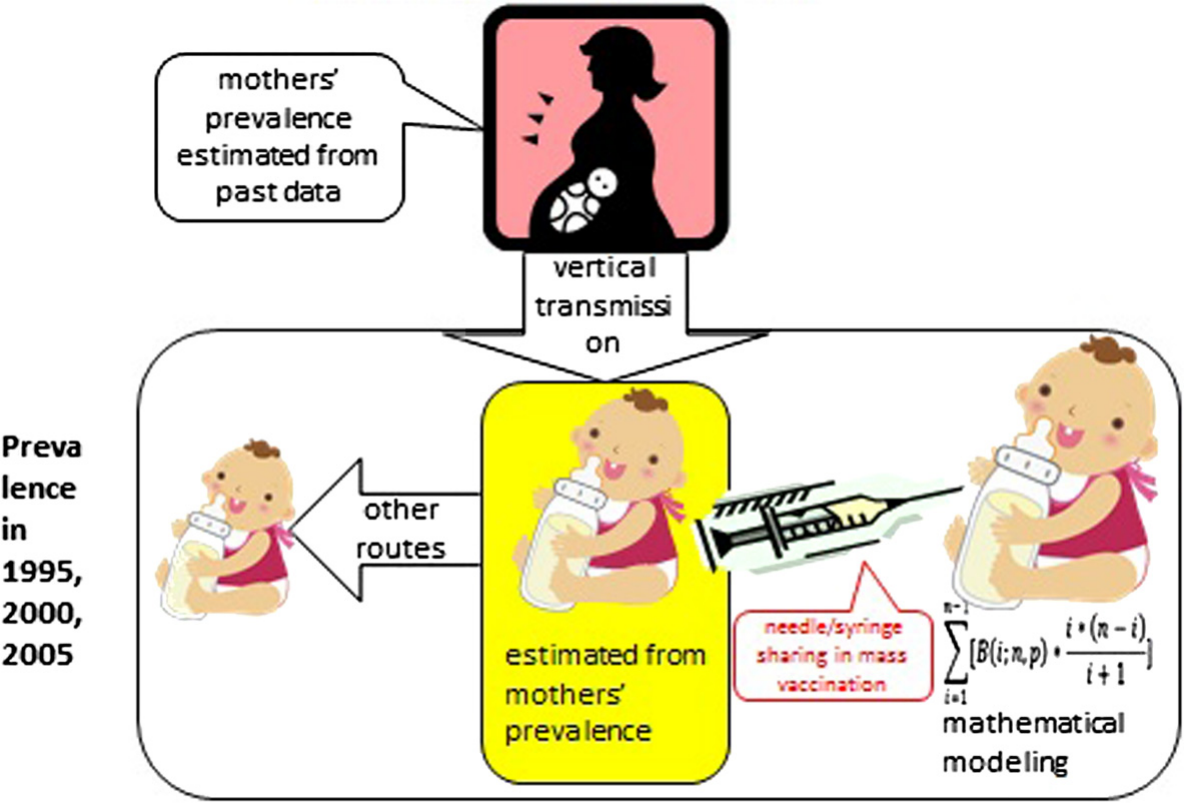
Modeling of HBV vertical and horizontal infection in children.

It is impossible to estimate exactly how many children were infected through needle/syringe sharing and how many through other routes. However, the mathematical model presented in this article is able to provide a rough estimate on the number of children infected through needle/syringe sharing in mass vaccination under estimated prevalence and given conditions (see Figure 6).

In case of a cohort born between 1951 and 1955, the model tells that the prevalence in 1995 (1.46%) would have been achieved if more than five children shared a needle/syringe four times given their estimated prevalence at birth (0.65%) and the probability of infection (10%). If such practice was plausible at that time, it would be reasonable to assume that most of the horizontal infection (1.46–0.65% = 0.81%) was attributable to mass vaccination.

However, if the prevalence of this cohort in 1995 was 5%, the model tells that 5% prevalence would not have been possible under the given conditions because it requires either more than 15 children to share a needle/syringe four times or more than ten children to share a needle/syringe five times, which does not appear plausible. Then one would assume that 3.54% (=5–1.46%) would have been infected through other routes (infection through needle/syringe sharing had a minor role compared with other routes).

The model is also useful for public health professionals to assess the extent of accidental infections in sporadic iatrogenic outbreaks still occurring in developing countries [20,21].

## Conclusions

Japan achieved success in controlling infectious diseases through mass vaccination programs in the postwar period. On the other hand, Japan also had a bitter experience in that many children were infected with HBV and became carriers due to the mass vaccination program. More than half of the HBV carriers born in the early 1950s might have contracted the disease through mass vaccinations.

In the absence of firm evidence, the author presented a mathematical model to estimate the extent of such iatrogenic infection under certain conditions.

Japan’s experience needs to be shared with other countries as a caution in conducting mass vaccination programs under scarce needle/syringe supply.

## Abbreviations

BCG: Bacillus Calmette-Guerin; HBV: Hepatitis B virus; HCV: Hepatitis C virus; HBsAg: Hepatitis B surface antigen; HBeAg: Hepatitis B e antigen; HIV: Human immunodeficiency virus; TST: Tuberculin skin test.

## Competing interests

The author has no competing interests to declare.

## Supplementary Material

Additional file 1 Multilingual abstracts in the six official working languages of the United Nations. Click here for file
