# Supplementary material for: A mathematical model to predict the risk of hepatitis B infection through needle/syringe sharing in mass vaccination
Source: Infect Dis Poverty. 2013 Nov 19;2:28. doi: 10.1186/2049-9957-2-28 (PMC4177201; doi:10.1186/2049-9957-2-28)
Supplement: Additional file 1 — Multilingual abstracts in the six official working languages of the United Nations. [file 2049-9957-2-28-S1.pdf]

Translation of the abstract into the six official working languages of the United Nations

نموذج رياضي للتنبؤ بخطر العدوى بالإلتهاب الكبدي الوبائ ب عن طريق تقاسم الإبر/الحُقن أثناء حملات التطعيم واسعة النطاق

إتسوجي أوكاموتو

الملخص

الخلفية: أقرت الحكومة اليابانية أن حالة التقاضي بسبب الإصابة بفيروس B إلتهاب الكبد الوبائي HBV - عن طريق الناقلين المصابين الذين يدعون أنهم أصيبوا بالعدوى من خلال تقاسم إبرة/حقنة أثناء حملات التطعيم واسعة النطاق - على اتفاق تغطية شاملة للتعويضات. ومع ذلك، فإنه من الصعب تقدير العدد الحالي من إصابات HBV الأفقية التي حدثت جراء حملات التطعيم واسعة النطاق، وكذلك كيفية حدوث إصابات HBV العمودية عن طريق العدوى من الأمهات.
الطرق: تم اقتراح وصياغة نموذجاً رياضياً للتنبؤ بخطر العدوى عن طريق تقاسم الإبر/حقنة أثناء حملات التطعيم واسعة النطاق. وتم تقديم هذه الصيغة على شكل رسم بياني لوغاريتمي يُمكن المستخدمين من حساب وتقدير الكيفية التي حدثت بها الإصابة لكثير من الناس عند إعادة استخدام أو تقاسم الإبر/الحُقن لعدة مرات واحتمالات عدوى مُعينة ومُتوقعة. تم تطبيق الصيغة على البيانات التاريخية لاختبارات الجلد (TSTs) واختبارات تطعيم BCG، ومن ثم تم تصميم صيغة أفضل لتقدير العدد المُستخدم الذي تم تقاسم الإبر/الحقن في الأفواج الميلادية المختلفة.
النتائج: بالنسبة للأفواج ممن وُلدوا بين عامي 1951 و 1955، كانت نسبة انتشار نقل HBV هي 0.65% - عند الولادة وذلك من خلال الانتقال العمودي، بينما تضاعف في عام 1995 وأصبح (1.46 ٪) وذلك عن طريق انتقال العدوى الأفقية. إذا افترضنا أن احتمال العدوى عن طريق تقاسم الإبر/الحقن كان 10٪، فمن المحتمل نظرياً أن يُصاب خمس أشخاص أو أكثر ممن يشتركون في إبرة/حقنة أربع مرات، مما حقق انتشار ناقلات الإلتهاب HBV في عام 1995. ومع ذلك، إن أصغر الأفواج ممن ولدوا بين عامي 1981 و 1985، لا تُذكر آثار تقاسم الإبر/الحقن آنذاك، وذلك لأن انتشار العدوى من ناقلات HBV كان أقل من معدل انتشار عند الولادة، في وقت لاحق.
الاستنتاجات: أكثر من نصف ناقلي إلتهاب HBV كانت ولادتهم في بداية الخمسينات وقد أصيبوا بالمرض عن طريق حملات التطعيم الواسعة. وعليه، يحتاج اليابان إلى تقاسم خبرته مع البلدان الأخرى، على أن تتوخى تلك البلدان منتهى الحذر في تنفيذ برامج وحملات التطعيم الواسعة والشاملة وتتخلى عن مُنطلق الشح في توريد الإبر/الحقن (291 كلمة).

Translated from English version into Arabic by Freelanceabunar, through

TRANSLATORS
WITHOUT BORDERS

用数学模型预测大规模疫苗接种重复使用针头/注射器感染乙肝病毒的风险

Etsuji Okamoto

摘要

引言：日本曾发生过一起因在疫苗接种计划中重复使用针头/注射器导致接种者感染乙型肝炎病毒（HBV）的集体诉讼案，该诉讼案经日本政府出面以一次性补偿协议获得解决。然而，在 HBV 携带者中，很难估计哪些是因大规模疫苗接种的水平感染，哪些是通过母婴传播的垂直感染。

方法：通过建立数学模型，预测大规模接种疫苗中重复使用针头/注射器而感染乙肝病毒的风险。开发的数学模型为对数曲线，通过该模型预测在某一特定的感染概率水平下，同一针头/注射器经一定人数和次数的重复使用后感染乙肝的人数。通过导入人群结核菌素皮试实验和卡介苗接种的历史数据，确定了不同出生队列中重复使用针头/注射器的最佳估计值。

结果：在 1951-1955 年出生队列中，母婴传播的乙肝病毒感染率为 0.65％，而 1995 年通过水平传播的感染率增加了一倍以上（1.46％）。假设重复使用针头/注射器感染乙肝病毒的概率为 10％，理论上，平均五人或以上共用针头/注射器四次的感染率可达到 1995 年的水平。然而，在 1981-1985 年出生队列中，由于乙肝病毒感染几率与年龄成反比，因而重复使用针头/注射器的影响可以忽略。

结论：20 世纪 50 年代初出生的乙肝病毒携带者中，有半数以上可能在大规模接种疫苗中感染了病毒。日本的这一经验应与其他国家分享，各国应从这一大规模疫苗接种计划中重复使用稀缺针头/注射器的事件中汲取经验。

Translated from English version into Chinese by Qian Ying-jun, through

CHINESE CENTER FOR DISEASE CONTROL AND PREVENTION
NATIONAL INSTITUTE FOR PARASITIC DISEASES

Modèle mathématique pour la prédiction du risque d'hépatite B par partage d'aiguille/de seringue au cours des vaccinations de masse

Etsuji Okamoto

Résumé

Contexte : Le gouvernement japonais a réglé par un accord de dédommagement global une plainte collective de porteurs du virus de l'hépatite B (HBV) qui affirmaient avoir été infectés par des aiguilles ou seringues partagées au cours de vaccinations de masse. Il est toutefois difficile d'estimer combien de porteurs actuels du HBV ont été infectés lors de vaccinations de masse (contamination horizontale) et combien ont été contaminés par leur mère (contamination verticale).

Méthodologie : Un modèle mathématique pour la prédiction du risque d'infection par le partage d'aiguilles ou de seringues au cours de vaccinations de masse a été proposé et une formule élaborée. Cette formule a été présentée dans un graphique logarithmique, de façon à permettre aux utilisateurs d'estimer le nombre de personnes qui pourraient être infectées en cas de partage d'aiguilles/de seringues par un nombre donné de personnes, sur un certain temps, avec une certaine probabilité d'infection. Elle a ensuite été appliquée aux données historiques obtenues lors de campagnes de dépistage de masse par IDR à la tuberculine et de vaccination par le BCG, afin de déterminer la meilleure estimation possible de la fréquence des partages d'aiguilles/de seringues dans les différentes cohortes de naissances.

Résultats : Pour la cohorte la plus âgée, née entre 1951 et 1955, la prévalence des porteurs du HBV, qui était de 0,65 % à la naissance par transmission verticale, avait plus que doublé en 1995 (1,46 %) par transmission horizontale. Si l'on suppose une probabilité d'infection par le partage d'aiguilles/de seringues de 10 %, il est théoriquement probable que cinq personnes ou plus, en moyenne, ont partagé une aiguille ou une seringue quatre fois pour atteindre la prévalence des porteurs de HBV de 1995. En revanche, pour la plus jeune cohorte, née entre 1981 et 1985, les effets du partage d'aiguilles/de seringues étaient négligeables, puisque la prévalence tardive des porteurs de HBV était inférieure à la prévalence à la naissance.

Conclusions : Plus de la moitié des porteurs de HBV nés au début des années 1950 ont peut-être contracté la maladie au cours de vaccinations de masse. L'expérience du Japon doit être partagée avec les autres pays, afin de mettre en garde ceux-ci contre le déploiement de programmes de vaccinations de masse lorsque les stocks d'aiguilles et de seringues sont insuffisants. (377 mots).

Translated from English version into French by Suzanne Assenatl, through

TRANSLATORS
WITHOUT BORDERS

Математическая модель предсказания риска заражения гепатитом В через общие шприцы/иглы во время массовых вакцинаций

Этсудзи Окамото

Аннотация

Проблема: Правительство Японии с помощью массовых компенсаций пришло к мировому соглашению с носителями вируса гепатита В (HBV), подавшими коллективный иск в адрес правительства, утверждая, что они заразились из-за использования общих игл/шприцев во время массовой вакцинации. Тем не менее, подсчитать, сколько человек, зараженных HBV в настоящий момент, получили инфекцию горизонтальным путем (в ходе массовых вакцинаций), а сколько – вертикальным (от матерей), довольно трудно.

Методы: Была предложена математическая модель и выработана формула предсказания риска передачи инфекции через общие иглы/шприцы в ходе массовых вакцинаций. Формула была представлена в виде логарифмического графика, который позволяет пользователям подсчитать, сколько человек будет заражено при использовании общего шприца или общей иглы, в зависимости от количества человек и случаев использования шприца или иглы, а также с учетом определенной вероятности заражения. Затем формула была применена к историческим данным о случаях массовой туберкулинодиагностики с помощью кожных проб (проб Манту) и массовой вакцинации БЦЖ, на основании которых был совершен оптимальный подсчет случаев использования общих игл/шприцев в разных возрастных когортах.

Результаты: В самой старшей когорте, рожденной между 1951 и 1955 гг., распространенность носителей HBV — 0.65% при рождении, зараженных вертикальным путем — к 1995 г. увеличилась более чем в два раза (и составляла 1.46%) из-за заражения путем горизонтальной передачи. Если принятая вероятность заражения через общие иглы/шприцы составляет 10%, теоретически возможно, что для получения распространенности носителей HBV в 1995 г., в среднем по пять или более человек использовали общую иглу/общий шприц четыре раза. Тем не менее, для самой младшей когорты, рожденной между 1981 и 1985 гг., эффект использования общих игл/шприцев ничтожно мал, поскольку дальнейшая распространенность носителей

HBV была меньше распространенности при рождении.

Выводы: Более половины носителей HBV, рожденных в начале 1950-х гг., могли заразиться гепатитом в ходе массовых вакцинаций. Об опыте Японии необходимо сообщить другим странам в качестве предостережения против проведения программ массовой вакцинации при недостаточном количестве игл/шприцев.

Translated from English version into Russian by Scylla, through

TRANSLATORS
WITHOUT BORDERS

Modelo matemático para anticipar el riesgo de contagio de la Hepatitis B a través de la reutilización de jeringas o agujas en vacunaciones masivas.

Etsuji Okamato.

Resumen

Contexto. Mediante un acuerdo de indemnización colectivo, el gobierno japonés puso fin a un litigio colectivo iniciado por los portadores del virus de la Hepatitis B (VHB, o *HBV* según sus siglas en inglés), quienes resultaron infectados en una vacunación masiva en la que se reutilizaron agujas/jeringas. No obstante, resulta difícil calcular qué cantidad de los portadores presentes contrajeron el VHB en forma horizontal a través de la vacunación masiva y cuántos a través de sus madres mediante transmisión vertical.

Métodos. Se propuso un modelo matemático para predecir el riesgo de infección mediante la reutilización de agujas/jeringas en vacunaciones masivas y, de esa manera, se desarrolló una fórmula. La fórmula se representó en un gráfico logarítmico que permite calcular qué cantidad de personas se infectaría si se reutilizaran las agujas/jeringas por qué cantidad de personas y cuántas veces bajo determinada probabilidad de infección. Más tarde, dicha fórmula se utilizó para los datos históricos de vacunación de la BCG y reacción de Mantoux, sobre los cuales se pudo realizar un cálculo más preciso con respecto a la cantidad de jeringas/agujas reutilizadas para diferentes cohortes de nacimiento.

Resultados. Para el cohorte conformado por las personas de mayor edad, nacidas entre los años 1951 y 1955, la prevalencia de portadores del VHB, con un 0,65% de contagio mediante transmisión vertical, fue superado en más del doble en 1995 (1,46%) por contagio mediante transmisión horizontal. Si las estadísticas arrojan que la probabilidad de infección a través de agujas/jeringas reutilizadas es de un 10%, es, teóricamente, probable que un promedio de 5 o más personas haya compartido aguja/jeringa unas cuatro veces, a los fines de obtener la prevalencia de portadores del virus VHB en el año 1995,. Sin embargo, para el cohorte correspondiente al grupo de personas más jóvenes, nacidas entre 1981 y 1985, los efectos de la reutilización de las agujas/jeringas resultaron insignificantes debido a que la prevalencia posterior de portadores del VHB fue inferior a la prevalencia en el momento de nacimiento.

Conclusiones. Más de la mitad de los portadores del virus de la Hepatitis B nacidos a comienzos de la década de 1950 podrían haberse contagiado dicha enfermedad a través de las vacunaciones masivas. Es importante que la experiencia de Japón sirva de ejemplo y se de a conocer en otros países a modo de prevención al momento de implementar programas de vacunación masivos en los que escaseen los insumos de agujas/jeringas.

Translated from English version into Spanish by VeroAnelli, through

TRANSLATORS
WITHOUT BORDERS
